# Functional Characterization of Laccase Isozyme (PoLcc1) from the Edible Mushroom *Pleurotus ostreatus* Involved in Lignin Degradation in Cotton Straw

**DOI:** 10.3390/ijms232113545

**Published:** 2022-11-04

**Authors:** Guoqing Li, Yahui Wang, Peilei Zhu, Guiyun Zhao, Caiyu Liu, Hongyuan Zhao

**Affiliations:** 1 State Key Laboratory of Horticultural Crop Germplasm Resources Creation and Utilization of Ministry of Agriculture and Rural Affairs, Institute of Horticulture Research, Anhui Academy of Agricultural Sciences, Hefei 230031, China; 2 College of Life Science, Anhui Agricultural University, Hefei 230036, China; 3 Provincial Resource Database of Wood Rot Edible Mushrooms in Anhui Province, Hefei 230031, China

**Keywords:** *Pleurotus ostreatus*, laccase, *Lacc1*, cotton stalk, lignin degradation, functional groups

## Abstract

Fungal laccases play important roles in the degradation of lignocellulose. In this study, the laccase producing cotton straw medium for *Pleurotus ostreatus* was optimized by single-factor and orthogonal experiments, and to investigate the role of *Lacc1* gene, one of the laccase-encoding genes, in the degradation of cotton straw lignin, an overexpression strain of *Lacc1* gene was constructed, which was analyzed for the characteristics of lignin degradation. The results demonstrated that the culture conditions with the highest lignin degradation efficiency of the *P. ostreatus* were the cotton straw particle size of 0.75 mm, a solid–liquid ratio of 1:3 and containing 0.25 g/L of Tween in the medium, as well as an incubation temperature of 26 °C. Two overexpression strains (OE L1-1 and OE L1-4) of *Lacc1* gene were obtained, and the gene expression increased 12.08- and 33.04-fold, respectively. The results of ^1^H-NMR and FTIR analyses of significant changes in lignin structure revealed that *Lacc1* gene accelerated the degradation of lignin G-units and involved in the cleavage of β-O-4 linkages and the demethylation of lignin units. These findings will help to improve the efficiency of biodelignification and expand our understanding of its mechanism.

## 1. Introduction

Laccase is a multi-copper oxidase that catalyzes the oxidation of various phenolic and non-phenolic substrates associated with the lignin structure, while reducing oxygen to water [1,2,3]. Due to the wide range of natural substrates, laccase is used in several fields of industrial and biotechnological applications, such as improvement of fiber properties, degradation of antibiotics and other pharmaceutical products, fuels, detoxification of environmental pollutants, stabilization of lignin by providing chemical precursors, and pulp bleaching paper industry [4,5,6,7,8]. Most of the known laccase enzymes originated from fungi, especially white rot fungi that can perform lignin degradation [9,10,11].

Fungal laccase is encoded by a multigene family and there are multiple isozymes that are mainly involved in physiological processes such as lignin degradation, fruiting body formation, pigment formation in asexual development and pathogenesis [12,13,14,15,16]. Jiao et al. [17] analyzed the expression patterns of 12 laccase encoding genes distributed in the genome of *P. ostreatus* PC15 strain and hypothesized that *PoLac2* might be closely related to lignin degradation by *P. ostreatus*. While *PoLac3* and *PoLac5* may be related to the formation of fruiting primordium and fruiting body, respectively, *PoLac12* may be related to the development of *P. ostreatus* fruiting body, and overexpression of *PoLac2* gene was found to enhance the laccase activity and lignin degradation of *P. ostreatus*. Lu et al. [18] performed qRT-PCR on 11 Laccase genes of *Volvariella volvacea*, and hypothesized that *Vvlcc3* was involved in stipe elongation by combining with heterologous expression in *Pichia pastoris*. Analysis of the transcriptome of different developmental stages and different tissues of *Flammulina velutipes* revealed that the expression of *lac4* gene was higher than other laccase genes at all developmental stages, suggesting it is involved in the biotransformation of lignin [19]. Successful overexpression of the *Pblac1* gene in white-rot fungus *Polyporus brumalis* revealed that the transformed strain exhibited higher lignin degradation activity and effective decolorization of RBBR dye compared to the wild type [20]. Homologous overexpression of the *Gtlcc3* gene in brown-rot fungus *Gloeophyllum trabeum* resulted in transformed strains with significantly higher lignin degradation and ethanol production than the wild type [21]. At present, the functional roles of laccase genes are mostly at the speculative stage, and the specific functional roles of each isoenzyme are not clearly defined, and there are fewer studies related to functional verification and molecular mechanisms.

Crop straw is mainly composed of cellulose, hemicellulose and lignin, in which cellulose molecules are embedded in a barrier formed by covalent bonding of lignin and hemicellulose, making it difficult for enzymes to contact with cellulose molecules, reducing the utilization and nutritional value of straw, which is the main obstacle to the effective conversion of lignocellulosic [22,23]. Cotton as one of the important economic crops in China, its harvest will also produce a large number of byproducts-cotton straw [24,25]. With the development of science and technology, urbanization and the improvement of living standards, the application of cotton straw as a living fuel decreases, and is burned in the fields by cotton farmers, both wasting resources and polluting the environment. On the contrary, the application of cotton straw as a base material has been further strengthened. However, cotton straw is similar to branch of tree, and compared with corn, rice and wheat straws, they have higher lignin content, which greatly limits the degradation by microorganisms [26].

In a previous study, it was found that *Pleurotus ostreatus* Suping 1 could be produced on cotton straw substrates [17], but the optimal production conditions and lignin degradation mechanism of cotton straw were not clear. Therefore, in this study, the optimal conditions for lignin degradation by *P. ostreatus* Suping 1 on cotton straw substrate were firstly screened by medium optimization experiments. Then, the *Lacc1* gene of *P. ostreatus* Suping 1 was obtained by homologous cloning, and the molecular mechanism of *Lacc1* in lignin degradation of cotton straw was analyzed by overexpression technique. The results of this study provide new insights into the mechanism of lignin degradation by white-rot fungi and provide useful guidance for the promotion and application of the laccase in *P. ostreatus*.

## 2. Results

### 2.1. Optimized Culture Factors for Laccase Production in P. ostreatus Suping 1

**Figure 1:**
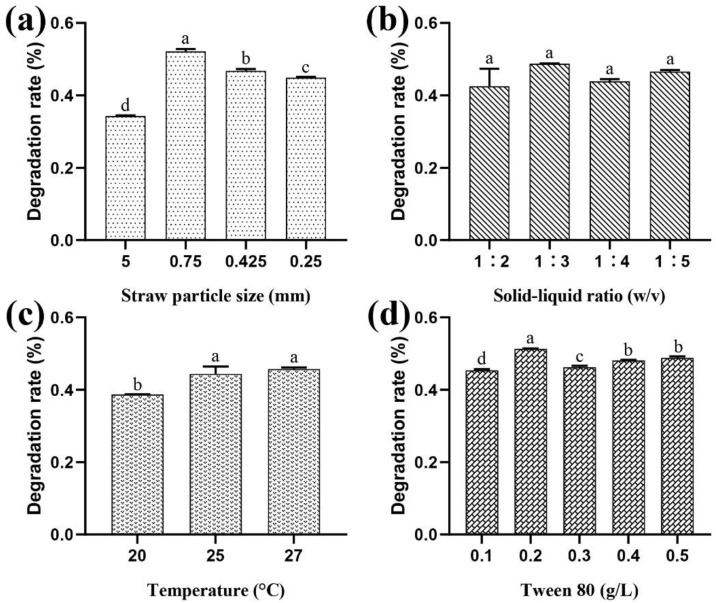
Single-factor test for screening the optimal conditions for lignin degradation in cotton straw by *P. ostreatus* Suping 1 with different (**a**) straw particle size, (**b**) solid–liquid ratio, (**c**) temperature and (**d**) concentration of Tween 80. a,b,c,d indicate significant differences among treatments (*p* ≤ 0.05).

The degradation rate of cotton straw lignin by *P. ostreatus* Suping 1 differed significantly (*p* > 0.05) among the four treatments with straw particle size of 5 mm, 0.75 mm, 0.425 mm and 0.25 mm. The highest degradation rate of cotton straw lignin was found at the straw particle size of 0.75 mm (Figure 1a). However, there was no significant difference (*p* > 0.05) in the effect of the ability of *P. ostreatus* Suping 1 to degrade cotton straw lignin in the four treatments of the solid–liquid ratio of 1:2, 1:3, 1:4 and 1:5 (Figure 1b). Among the three temperature experimental groups at 20 °C, 25 °C, and 27 °C, the degradation rate of cotton straw in the 25 °C experimental group differed significantly from that in the 20 °C experimental group (*p* < 0.05) and not significantly different from that in the 27 °C experimental group (*p* > 0.05), and the degradation rate of cotton straw at 27 °C was slightly higher than that at 25 °C, indicating that the ability of *P. ostreatus* Suping 1 to degrade lignin increased with the increase in temperature in a certain temperature range (Figure 1c). The treatment groups with the addition of surfactant Tween 80 concentration of 0.1 g/L, 0.2 g/L, 0.3 g/L, 0.4 g/L and 0.5 g/L showed significant differences in the degradation rate of cotton straw, except for Tween 80 at concentrations of 0.4 g/L and 0.5 g/L (*p* > 0.05), and *P. ostreatus* Suping 1 degraded cotton straw lignin the most at the Tween 80 content of 0.2 g/L (Figure 1d).

Based on the results of the Single-factor optimization experiments, nine different formulations were prepared, and the experimental runs were carried out based on the experimental matrix, and the observed responses are shown in Table 1. The laccase activity of *P. ostreatus* Suping 1 was significantly different under different combinations of factors. No. 5, which was under the multifactorial conditions of straw particle size of 0.75 mm, solid–liquid ratio of 1:3, incubation temperature at 26 °C and Tween content of 0.25 g/L, had the highest laccase activity of 125.65 ± 8.36 U/mL, which was significantly stronger than other culture conditions. This indicates that the laccase production ability of *P. ostreatus* Suping 1 is stronger under No. 5 culture condition. Among the four factors to be investigated, in descending order of importance, the effect on the laccase activity of *P. ostreatus* Suping 1 was B > A > C > D, which means that the most influential factor was the solid–liquid ratio, followed by the straw particle size, again by the incubation temperature, and the least influential was the Tween 80 content (Table 2).

### 2.2. Cloning and Analysis of the Laccase-Encoding Gene (Lacc1) in P. ostreatus Suping 1

**Figure 2:**
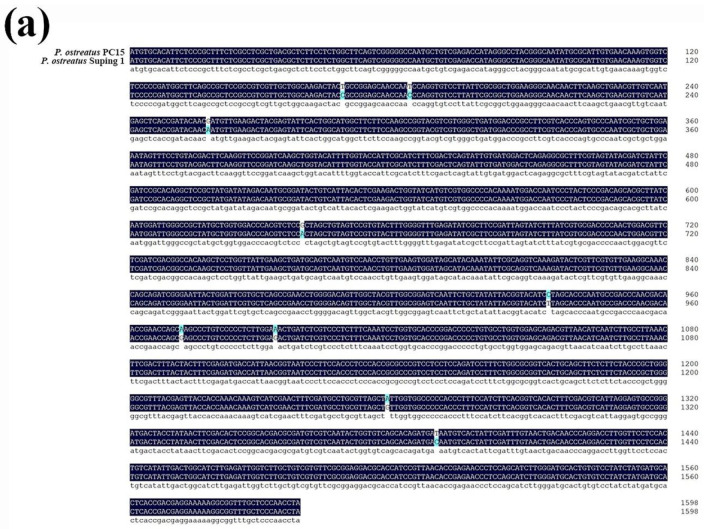

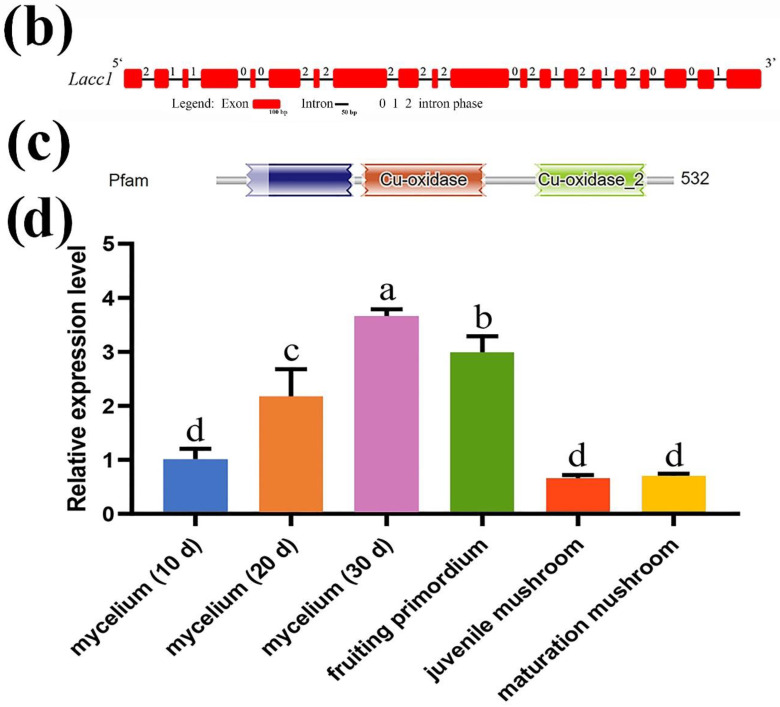
The characterization and expression pattern in *P. ostreatus* Suping 1 of laccase-encoding gene (*Lacc1*). (**a**) CDS sequence alignment of *Lacc1* between *P. ostreatus* PC15 and *P. ostreatus* Suping 1; (**b**) gene structure of *Lacc1* in *P. ostreatus* Suping 1; (**c**) the Pfam domain matches and features of *Lacc1* in *P. ostreatus* Suping 1; (**d**) the expression pattern of *Lacc1* gene in different growth stages of *P. ostreatus* Suping 1. Different letters above the chart columns indicate significant differences among treatments (*p* ≤ 0.05).

In the genome of *P. ostreatus* Suping 1, the full-length cDNA sequences of *Lacc1* gene were 1599 bp. By comparison, the CDS sequence of the *Lacc1* gene in strain *P. ostreatus* Suping 1 differed from that in strain *P. ostreatus* PC15 by nine nucleotides (Figure 2a). DNA sequence analysis showed that 20 exons were interrupted by 19 introns in *Lacc1* of *P. ostreatus* Suping 1 (Figure 2b) and have three Cu-oxidase domains (Figure 2c). The mRNA expression level of *Lacc1* gene in mycelial period (10 d, 20 d and 30 d), fruiting primordium, juvenile mushroom and maturation mushroom of *P. ostreatus* Suping 1 was detected by qRT-PCR, and the results showed that the expression of *Lacc1* gene increased and then decreased in these culture stages of *P. ostreatus* Suping 1, and the highest expression was in mycelial period (30 d), followed by fruiting primordium (Figure 2d). This indicates that it may perform physiological functions more in the mycelium and fruiting primordium stage.

### 2.3. Overexpression of Lacc1 Gene

**Figure 3:**
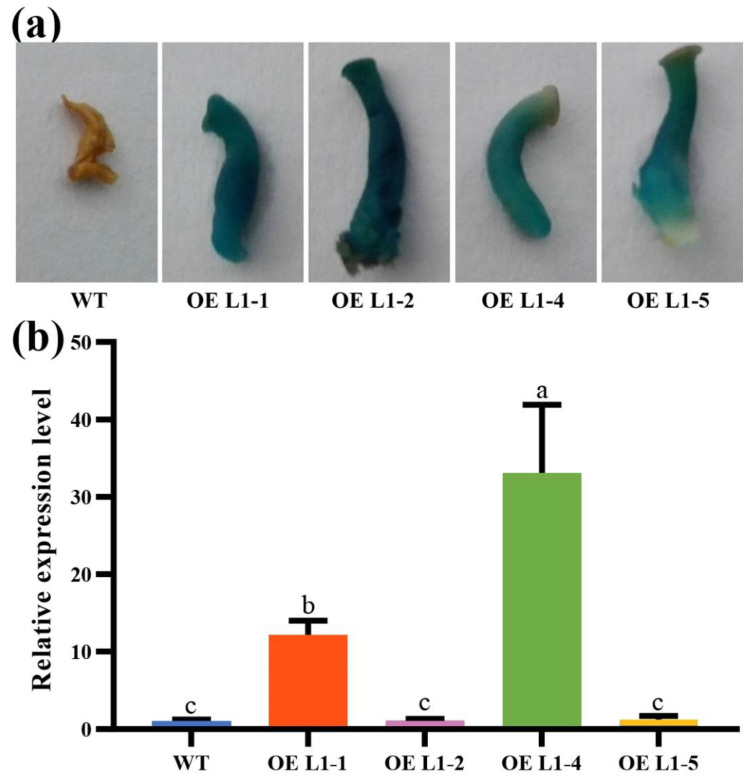
Overexpression of *Lacc1* gene in *P. ostreatus* Suping 1. (**a**) GUS analysis of *Lacc1* gene overexpression transformants; (**b**) gene-relative transcript abundance of wild type (WT) and transformants (OE L1-n) after cultivating in the cotton straw medium for 30 days. Different letters above the chart columns indicate significant differences among treatments (*p* ≤ 0.05).

To investigate the lignin degradation function of the *Lacc1* gene, the CDS sequence of the Lacc1 gene from *P. ostreatus* Suping 1 was cloned into pCAMBIA1304 and transformed into *P. ostreatus* Suping 1 using the ATMT method, driven by the *Pogpd* promoter. Subsequently, four transformants were randomly selected to verify whether the reporter gene β-glucosidase (*gus*) was inserted into the genome of *P. ostreatus* Suping 1. The results showed that these mutants showed the presence of the amplification product of *gus*. In addition, GUS histochemical assays showed that after 10 h of staining, the immature substrates of the mutants turned blue in the buffer of GUS staining, whereas the wild type did not, indicating that GUS was expressed in the mutants. The results indicated that the exogenous *Lacc1* gene had been successfully integrated into the genome of the *P. ostreatus* Suping 1 (Figure 3a). After cultivating in the cotton straw medium for 30 days, the expression of *Lacc1* gene in each transformant was detected, in which the expression of *Lacc1* gene in OE L1-1 and OE L1-4 was 12.08 and 33.04 times higher than that in the wild-type strain, respectively (Figure 3b).

### 2.4. Characterization of the Function of Lacc1 Gene in Lignin Degradation of Cotton Straw

**Figure 4:**
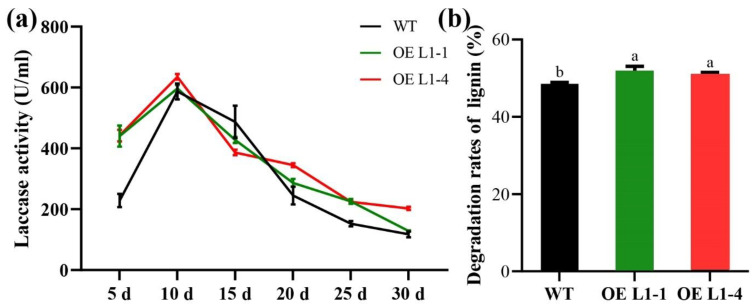
Laccase activity and lignin degradation rates of overexpressed transformants. (**a**) Evolution of the laccase activity of wild-type (WT) and *Lacc1* gene overexpressed transformants (OE L1-1 and OE L1-4) of *P. ostreatus* Suping 1 in cotton straw medium; (**b**) lignin degradation rates of wild type and transformants after cultivating in the cotton straw medium for 30 days. Different letters above the chart columns indicate significant differences among treatments (*p* ≤ 0.05).

To clarify the function of the *Lacc1* gene in lignin degradation of cotton straw, wild-type and overexpressed *P. ostreatus* Suping 1 were cultured in cotton straw medium and their laccase activity was measured at days 5, 10, 15, 20, 25 and 30, as well as their ability to degrade cotton straw lignin (30th day). The overexpressed transformants OE L1-1 and OE L1-4 secreted higher extracellular laccase activity than the wild type, except on the 15th day when the extracellular laccase activity was lower than that of the wild type, especially on the 5th day of incubation which was significantly higher than that of the wild type, with an approximately 1-fold increase in laccase activity (Figure 4a). The lignin degradation rate of the wild-type strain was 48.56 ± 0.23% for cotton straw. The lignin degradation rates of overexpressed transformants were 51.89 ± 0.94% (OE L1-1) and 51.10 ± 0.31% (OE L1-4), respectively (Figure 4b). The lignin degradation rates of the overexpressed transformants were increased by 6.86% (OE L1-1) and 5.23% (OE L1-4), respectively, compared to the wild type. Thus, it was direct evidence that *Lacc1* gene was involved in the biodegradation of cotton straw lignin.

### 2.5. FTIR Analysis of the Degradation Products from Lignin

**Figure 5:**
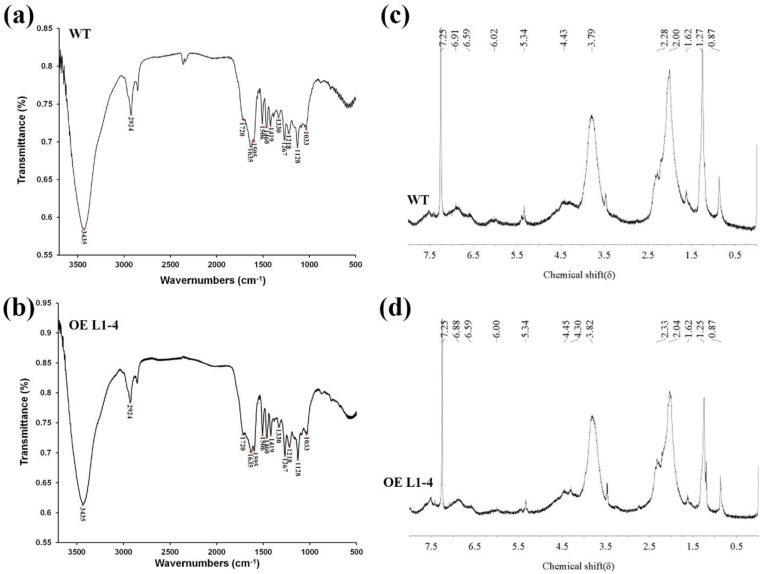
Infrared spectroscopy and ^1^H NMR analysis of lignin degradation products from cotton straw medium inoculated with wild-type (WT) and *Lacc1* gene overexpressed transformants (OE L1-4) of *P. ostreatus* Suping 1 for 30 days. (**a**) Infrared spectroscopy analysis of cotton straw medium –inoculated with wild-type strain; (**b**) Infrared spectroscopy analysis of cotton straw medium inoculated with OE L1-4 strain; (**c**) ^1^H NMR analysis of cotton straw medium inoculated with wild-type strain; (**d**) ^1^H NMR analysis of cotton straw medium inoculated with OE L1-4 strain.

To explore the molecular mechanism of *Laac1* gene involved in lignin degradation. Lignin was extracted from cotton straw medium inoculated with OE L1-4 and WT for 30 days, and the structural changes in lignin degradation were determined using infrared spectroscopy to resolve the characteristics of absorption peaks and corresponding functional groups in each infrared spectrogram (Figure 5a, b). The intensity of the absorption peak of each functional group is the intensity ratio to that of the absorption peak at 1508 cm^−1^. The absorption peak at 2924 cm^−1^ was mainly C-H stretching in methyl and methylene groups, where the relative absorption intensity of lignin in the culture medium of OE L1-4 was 0.75, and the relative absorption intensity of lignin in the culture medium of wild-type strain was 0.95. The reduced intensity of lignin in the culture medium of OE L1-4 indicates that the *Lacc1* gene may promote the demethylation/demethoxylation reaction of the methyl/methoxy on the benzene ring of the lignin monomer. The absorption peak at 1635 cm^−1^ corresponds to the C=O stretching in conjugated aryl ketene of carbonyl groups, and the relative absorption intensity at 1635 cm^−1^ of lignin from culture medium of wild-type strain was higher than that of culture medium inoculated with OE L1-4, indicating that overexpression of the *Lacc1* gene reduced the carbonyl conjugated aromatic ketone C=O-related reaction of lignin (e.g., production of phenolic hydroxyl groups, etc.). The absorption peaks at 1595 cm^−1^ and 1506 cm^−1^ corresponded to the Aromatic skeletal vibrations plus C=O stretching and Aromatic skeletal vibrations, respectively, and the cultures inoculated with wild-type and OE L1-4 strains had comparable relative intensities at both absorption peaks, indicating that the *Lacc1* gene was not affecting the opening and degradation of the benzene rings in lignin (Table 3).

### 2.6. ^1^H-NMR Analysis of the Degradation Products from Lignin

NMR hydrogen spectroscopy has also been applied to determine structural changes in lignin degradation (Figure 5c, d and Table 4). Based on the ^1^H-NMR spectrogram for the acetylated lignin, the lignin range was distinguished, and linin was integrated to calculate the proton ratio between functional groups and bond-type structures [28]. The percentage of Aromatic protons in guaiacyl units of acetylated lignin in cotton straw medium cultured with OE L1-4 was decreased to 0.64 (CDCl_3_ was 1) compared to wild type (0.68). This indicates that the *Lacc1* gene accelerates the degradation of G-unit lignin. However, the percentage of Aromatic protons in syringyl units of acetylated lignin in cotton straw medium cultured with OE L1-4 was increased to 0.45 (CDCl_3_ was 1) compared to wild type (0.35). It is hypothesized that the *Lacc1* gene affects the degradation of S-unit lignin. The percentage of acetylated lignin Hα of β-O-4 and β-1 structures and Hγ and Hβ of β-O-4 structures in cotton straw medium inoculated with OE L1-4 after 30 days of incubation was higher than that of the wild type, whereas the percentage of Hα of β-β structures was lower than that of the wild type, indicating that *Lacc1* gene is involved in the breaking of chemotactic bonds in β-β structures. Compared with the wild type, the cotton straw medium inoculated with OE L1-4 showed a significant increase in the proportion of H of aromatic acetates for acetylated lignin, whereas its proportion of H of aliphatic acetates showed a significant decrease. This indicates that the *Lacc1* gene is involved in the production of phenolic hydroxyl substances and the reduction in alcoholic hydroxyl substances in lignin degradation.

## 3. Discussion

Studies have shown that the substrates cellulose: lignin ratios were found to be positively correlated to mycelial growth rates and to mushroom yield of *P. ostreatus* [30,31], and cotton straw is a substrate with high lignin content. In reports on cotton straw culture of edible mushrooms, it was found that higher yields could be obtained by applying to cotton straw culture of edible mushrooms relative to other crop substrates [32,33,34,35], in which lignin degrading enzymes played a crucial role [36,37,38]. In this study, we first screened the optimum culture conditions for laccase enzyme activity of *P. ostreatus* Suping 1. The final culture conditions for the highest lignin degradation efficiency of the *P. ostreatus* Suping 1 were determined by single-factor and orthogonal experiments: the size of cotton straw particle size in the medium was 0.75 mm, the medium had a solid–liquid ratio of 1:3, and contained 0.25 g/L of Tween, as well as the incubation temperature was 26 °C.

Two overexpression strains (OE L1-1 and OE L1-4) of *Lacc1* gene were obtained by *Agrobacterium tumefaciens*-mediated method, and the gene expression increased 12.08 and 33.04-fold at day 30, respectively, and the enzyme activity of OE L1-4 strain increased 71.05% at day 30 compared to WT strain, whereas the enzyme activity of OE L1-1 strain was not significantly different from WT strain, showing the difference between gene expression level and gene product yields, probably due to the time required for gene translation into protein [39,40]. The lignin degradation rate of the medium was increased by 6.86% and 5.23% for OE L1-1 and OE L1-4 strains, respectively, and there was inequivalence between the lignin degradation ability and the increase in the fold to gene expression, probably due to the different mRNA translation efficiency of the two overexpression strains [41,42].

The lignin in cotton straw were characterized by wet chemistry (carbohydrate analysis) and spectroscopy methods (FT-IR, 13C and 1H-13C HSQC NMR spectroscopy) as well as gel permeation chromatography (GPC), which showed that the lignin in cotton straw belonged to typical G-S lignin, mainly composed of G-type units (59%) and distinct S-type units (40%), and the inter-unit linkages were mainly composed of β-O-4’ (75.6%) and β-β’ (12.2%) [43]. To investigate the details of lignin biodegradation, Dong et al. used FTIR with CP/MAS13C -NMR to study the characteristics and process of sugarcane bagasse degradation by three lignin degrading fungi, *Phanerochaete chrysosporium* PC2, *Lentinula edode* LE16 and *P. ostreatus* PO45, and found that all three strains preferentially degraded the S-unit of sugarcane bagasse lignin [44]. In contrast, Zhang et al. used FTIR and 2D HSQC NMR to determine the lignin composition of degraded sacrau poplar and found that the G-unit lignin was more susceptible to degradation by *Trametes pubescens* C7571 and *T. versicolor* C6915 than the S-unit lignin, and found that the cleavage of β-O-4 linkages and the degradation of β-5 and β-β linkages clearly occurred [27]. It is observed that the preferential degradation of S- or G-units in lignin, as well as the cleavage of linkages of different bond types, is related to the strain class. In this study, the G/S of acetylated lignin in cotton straw after 30 days of incubation with the wild-type strain was 1.94, and the G/S of acetylated lignin in cotton straw with OE L1-4 was 1.42. Compared with the wild-type control, the G/S of acetylated lignin in the overexpressed strain was reduced, indicating that the *Lacc1* gene accelerates the degradation of lignin G-units. In addition, the G/S ratio of acetylated lignin in cotton straw medium inoculated with OE L1-4 strain was 1.42, which was lower than that of acetylated lignin in cotton straw medium inoculated with wild-type strain (1.94), but still higher than that of original acetylated lignin (1.20). It indicates that the *Lacc1* gene only slowed down the degradation of S-unit and accelerated the degradation of G-unit, but the degradation of S-unit was still faster than that of G-unit, that is, overexpression of *Lacc1* gene did not change the preferential degradation of S-unit in cotton straw lignin by *P. ostreatus*. Meanwhile, the *Lacc1* gene was found to be involved in the cleavage of β-O-4-type linkages.

From the ratio of H proton of aromatic acetates content to H proton of aliphatic acetates content, the ratio of phenolic to alcoholic hydroxyl groups in lignin can be obtained [29]. The ratio of phenolic to alcoholic hydroxyl groups in the acetylated lignin of cotton straw after 30 days of incubation with wild-type strains was 0.23, and the ratio of phenolic to alcoholic hydroxyl groups in the acetylated lignin of cotton straw of OE L1-4 increased to 0.36. It was demonstrated that the degradation of lignin by the *Lacc1* gene was mainly reflected in the demethylation of lignin units and thus promoting the depolymerization of the units, which is consistent with the inference that the degradation of lignin by *Lacc1* may be mainly reflected in the demethylation of lignin units rather than the oxidative breaking of the chemical bonds between the lignin benzene ring structures [45].

## 4. Materials and Methods

### 4.1. Strains

The *Pleurotus ostreatus* Suping 1 strain was provided by Vegetable Research Institute, Jiangsu Academy of Agricultural Sciences and preserved in the Institute of Horticulture, Anhui Academy of Agricultural Sciences. The *Escherichia coli* DH5α and *Agrobacterium tumefaciens* EHA105 strain were preserved in the laboratory. The pCAMBIA1304-*Pogpd*-*PoLac1* plasmids were constructed with the pCAMBIA1304 plasmids also kept in the same laboratory.

### 4.2. Single-Factor Optimization of Lignin Degradation in Cotton Straw by Pleurotus ostreatus

**Straw particle size**. Five grams of cotton straw with particle sizes of 5 mm, 0.75 mm, 0.425 mm and 0.25 mm were weighed into culture flasks, 22 mL of nutrient solution was added and autoclaved at 121 °C for 30 min. The nutrient solution was prepared by ammonium tartrate (22.0 g/L), macronutrients (20 g/L KH_2_PO_4_, 13.8 g/L MgSO_4_·7H_2_O, 1.0 g/L CaCl_2_, 0.6 g/L NaCl), trace elements (0.35 g/L MnSO_4_·H_2_O, 60 g/L FeSO_4_·7H_2_O, 110 mg/L CoCl_2_·6H_2_O, 60 mg/L ZnSO_4_·7H_2_O, 95 mg/L CuSO_4_·5H_2_O, 6 mg/L AlK(SO_4_)_2_·12H_2_O, 6 mg/L H_3_BO_3_, 6 mg/L Na_2_MoO_4_·2H_2_O), VB_1_ (100 mg/L) and water in the ratio of 1:15:15:3:16. Three *P. ostreatus* Suping 1 pieces of about 1 cm in diameter from PDA medium (boiled juice of 200 g/L potato, 20.0 g/L glucose, and 15.0 g/L agar added to solid medium) were inoculated into each culture flask and incubated at 25 °C for 20 days in the dark to determine the remaining medium lignin content.

**Solid–liquid ratio**. Five grams of cotton straw with particle sizes of 0.425 mm were weighed into culture flasks and 10 mL, 15 mL, 20 mL and 25 mL of nutrient solution were added to each treatment to make the solid–liquid ratio (*w*/*v*) 1:2, 1:3, 1:4 and 1:5, respectively, and autoclaved at 121 °C for 30 min. Different treatments were inoculated with *P. ostreatus* Suping 1 and the remaining medium lignin content was measured after 20 days of dark incubation at 25 °C.

**Temperature**. Five grams of cotton straw with a particle size of 0.425 mm were weighed into the culture flask, 22 mL of nutrient solution was added, sterilized and inoculated with *P. ostreatus* Suping 1. Then, the remaining lignin content of the medium was measured after 20 days of incubation under dark conditions at 20 °C, 25 °C and 27 °C, respectively.

**Tween**. Five grams of cotton straw with a particle size of 0.425 mm were weighed into the culture flask and 22 mL of nutrient solution containing different concentrations of Tween 80 was added, with final concentrations of Tween 80 are 0.1 g/L, 0.2 g/L, 0.3 g/L, 0.4 g/L and 0.5 g/L, respectively, and inoculated with *P. ostreatus* Suping 1 after sterilization. Then, after 20 days of culture at 25 °C in the dark, the remaining lignin content in the medium was measured.

### 4.3. Orthogonal Array Optimization of Lignin Degradation in Cotton Straw by P. ostreatus

The orthogonal experiment was performed with four factors of straw particle size (A), solid–liquid ratio (B), incubation temperature (C) and Tween content (D), and three levels of each factor were selected, as in Table 5, using orthogonal test L9 (3^4^) with nine treatments, each treatment was inoculated with five bottles and replicated three times. After 20 days of incubation, five bottles of medium were mixed and tested for lignin content, and the lignin degradation rate of *P. ostreatus* Suping 1 was statistically calculated, and the effect of each treatment factor on the lignin degradation rate of *P. ostreatus* Suping 1 was analyzed by ANOVA and Duncan’s multiple comparisons using SPSS version 17.0 statistical analysis software.

### 4.4. Determination of Lignin Content

After culture of *P. ostreatus* Suping 1 strains, the solid residue substrates were dried in an oven at 60 °C to a constant weight. 100 mL of acid washing buffer (4 mol/L HCl) was used to digest 1 g of the dried substrate residue for 60 min. The samples were then washed with acetone and petroleum ether using a cold leaching device. Subsequently, the sample was washed with 12 mol/L sulfuric acid solution for 3 h. Then, the sample was washed to neutral and dried in a vacuum oven at 130 °C to a constant weight. The dried samples were ashed in a muffle furnace at 550 °C for 2 h. After cooling, the samples were weighed and the lignin content in the substrate residue was calculated [46].

$$\mathrm{Lignin}\;\mathrm{content}\;\left(\%\right)=\frac{\mathrm{Weight}\;\mathrm{of}\;\mathrm{residue}\;\mathrm{after}\;\mathrm{drying}\;\mathrm{at}\;130\;{}^{\circ}C\left(g\right)-\mathrm{Weight}\;\mathrm{of}\;\mathrm{residue}\;\mathrm{after}\;\mathrm{ashing}\;\mathrm{at}\;550\;{}^{\circ}C\;\left(g\right)}{1\;g}$$

### 4.5. Optimization of Laccase Production by Orthogonal Array Method

Orthogonal array design was adopted for four culture conditions using Minitab 16 software to evaluate the factors influencing the yield of laccase. Each independent variable was tested at three levels such as high, middle, and low level. The symbol code and actual level of the variables and the experimental design are shown in Table 2. Four factors such as straw particle size, solid–liquid ratio, temperature, and Tween contents were studied in 9 experiments to calculate the standard error. The triplicate verification test was performed to check the optimum condition and the average value was taken as the response.

### 4.6. Laccase Activity Assay

The samples were removed every 5 days and 15 mL of purified water was added in batches and extracted overnight at 4 °C. Thereafter, shaking extraction was performed at 200 r/min for 1 h, followed by freezing centrifugation at 12,000 r/min for 10 min. Finally, the supernatant was obtained as the crude enzyme solution. The laccase activity was calculated by examining the oxidation of 2,2-azobis-(3-ethylbenzothiazoline-6-sulfonic acid) (ABTS) at 420 nm. One unit of activity was defined as the amount of enzyme converting 1 μmol of substrate per minute [47].

### 4.7. Cloning and Expression Pattern Analysis of Lacc1 in P. ostreatus Suping 1

The genomic DNA and RNA of *P. ostreatus* Suping 1 were extracted using the EasyPure^®^Plant Genomic DNA Kit (Trans, Beijing) and RNAprep Pure Plant Kit (Tiangen, China), respectively. Subsequently, the CDS sequence of LACC1 (KDQ27217.1) from strain *P. ostreatus* PC15 (GCA_000697685.1) submitted to NCBI was used to design primers (Lac1-F and Lac1-R, Table 6) to amplify the *Lacc1* gene sequence of *P. ostreatus* Suping 1 using the DNA and cDNA of *P. ostreatus* Suping 1 as templates, respectively. The amplified PCR products were purified, ligated to the pMD18-T vector (Takara, Dalian) and sequenced. The CDS sequence of *Lacc1* gene of *P. ostreatus* Suping 1 was used to design qRT-PCR primers (Lac1-qF and Lac1-qR) to detect the expression pattern of *Lacc1* gene in different growth stages of *P. ostreatus* Suping 1. RNA reverse transcription was performed using TransScript^®^ One-Step RT-PCR SuperMix (Trans, China) and qRT-PCR was performed using the Bio-rad Cfx96 Touch™ Deep Well Real-Time PCR detection system. The PCR volume was 20 μL, and *cyph* (transcript ID: 1058252) was set as the reference gene [48]. Three parallel replicates were set for each sample. Finally, relative gene expression was calculated by the 2^−ΔΔCt^ method. The reactions were contained in the following: 10 μL of TransStart Tip Green qRT-PCR SuperMix (2×) (Trans, China), 2 μL of template cDNA, 0.8 μL of forward and reverse primers and ddH_2_O to 20 μL. The PCR amplification conditions were performed as follows: 98 °C for 2 min, followed by 40 cycles of 98 °C for 10 s, 60 °C for 10 s and 68 °C for 30 s.

### 4.8. Agrobacterium Tumefaciens-Mediated Transformation of P. ostreatus

The glyceraldehyde-3-phosphate dehydrogenase (*gpd*) promoter was cloned from *P. ostreatus* [49]. Then, it was inserted into the expression plasmids pCAMBIA1304 to replace its CAMV35S promoter by using the restriction sites of *Hind* III and *Nco* I. Subsequently, *Lacc1* were cloned from *P. ostreatus* Suping 1 using specific primers (Lac1-eukF and Lac1-eukR, Table 5), respectively. The PCR products were digested with *Bgl* II and *Spe* I (Takara), then inserted into the pCAMBIA1304-*Pogpd* vector. Final vector plasmids were designated as pCAMBIA1304-*Pogpd*-*PoLac1*.

The *A. tumefaciens* strains EHA105, harboring pCAMBIA1304-*Pogpd*-*PoLac1*, were cultivated at 28 °C in LB medium (containing antibiotics kanamycin and rifampicin) to an OD600 of 0.6–0.8. Bacterial cells were then collected, centrifugated, and suspended in an induction medium (IM, boiled juice of 200 g L^−1^ potato, 20.0 g L^−1^ glucose, and including 200 μmol/L acetosyringone) to an OD600 of 0.5–0.6, pH 5.5, and the virulence of *A. tumefaciens* was induced by shaking at 150 rpm for 6 h at 28 °C.

The mycelia masses (0.9 × 0.9 cm) of *P. ostreatus* were grown on PDA for 7 days was immersed in pre-inducing bacterial culture for 30 min and then placed on solid induction medium for 4 days. Subsequently, the co-cultured mycelia were transferred to PDA with 100 μg/mL hygromycin and 300 μg/mL cefotaxime. The stability of the transformation was confirmed by subculturing colonies onto selective medium three times, then cultured on PDA for three generates to rejuvenate mycelia [50].

### 4.9. PCR Analysis and Visual Detection of β-Glucuronidase (GUS)

The genomic DNA of the putative transformants was extracted using the EasyPure^®^Plant Genomic DNA Kit (Trans, China). To confirm that the gene had been integrated into the genome, primers GUS-F and GUS-R (Table 1) for the *GUS* gene were used to verify whether the T-DNA was inserted by PCR (conditions: 94 °C for 3 min, followed by 35 cycles of amplification, 94 °C for 30 s, 59 °C for 30 s, 72 °C for 100 s, and ending after 10 min at 72 °C). To verify the expression of the introduced *GUS* reporter gene, the presumptive transformants were detected with the GUS Histochemical Assay Kit (MKbio, Shanghai).

### 4.10. Fourier Transform Infrared Spectroscopy (FTIR) Analysis

After cultivating for 30 days with the wild type and transformants, cotton stalks were removed and dried at 40 °C. According to the previous methods [51], milled wood lignin was extracted and purified. FTIR analysis was performed to determine the functional groups converted during the degradation of lignin by *Lacc1*-encoded laccase. Local microscopic FTIR with a KBr wafer was used, for which the milled wood lignin samples are homogeneous and representative. The micro-FTIR spectra of local areas of the wafer specimen were measured using a NICOLET iN10 MX spectrometer (Thermos Nicolet Corporation, Madison, WI, USA); connected to a Nicolet NicPlan IR microscope and an MCT detector. The spectral range was taken from 4000 to 650 cm^−1^ at a resolution of 4 cm^−1^.

### 4.11. ^1^H-NMR

Lignin was extracted from the uncultured substrates and the substrates that were cultured with *P. ostreatus* after 30 days. A total of 20 mg of milled wood lignin samples was weighed and dissolved in 2 mL of pyridine: acetic anhydride (1:1) mixture. Nitrogen was charged into the reaction bulb, which was placed in the dark at room temperature for 72 h. After the reaction was completed, the reactant was dripped into diethyl ether until precipitate was formed, after which the solution was centrifuged to separate the precipitate. The precipitate was then washed with diethyl ether six to eight times to remove the pyridine odor from the precipitate. Finally, the completely acetylated lignin was obtained. The acetyl-treated lignin samples were dissolved in 0.5 mL of DMSO, with tetramethylsilane as the internal standard. Finally, the ^1^H-NMR assay was conducted with a Bruker-400 superconducting NMR spectrometer (Bruker, Fällanden, Switzerland) at a frequency of 400 MHz [44].

## Figures and Tables

**Table 1:** Experimental matrix and response values from randomized runs in orthogonal array.

Run	Code Variable Level				Real Variable Level				Laccase Activity (U/mL) *
	A	B	C	D	Straw Particle Size (mm)	Solid–Liquid Ratio (*w*/*v*)	Temperature (°C)	Tween (g/L)	
1	3	3	2	3	1	1:4	27	0.25	97.8 ± 2.2 c
2	2	1	2	2	0.75	1:2	27	0.2	12.3 ± 0.9 e
3	1	3	1	2	0.5	1:4	26	0.2	50 ± 0 cd
4	3	1	1	1	1	1:2	26	0.15	31.6 ± 2.2 e
5	2	2	1	3	0.75	1:3	26	0.25	125.6 ± 8.4 a
6	3	2	3	2	1	1:3	28	0.2	80.4 ± 1.5 cd
7	1	1	3	3	0.5	1:2	28	0.25	11.1 ± 1.9 e
8	1	2	2	1	0.5	1:3	27	0.15	57.9 ± 1.1 d
9	2	3	3	1	0.75	1:4	28	0.15	115.3 ± 1.0 b

* Different letters in the table indicate significant differences among treatments (*p* ≤ 0.05).

**Table 2:** Analysis of different factors and levels on the degradation of cotton straw lignin by *P. ostreatus*.

Factors	Level			R	Significance
	K1	K2	K3		
A: Straw particle size (mm)	5.57 ± 0.18	9.43 ± 0.78	7.34 ± 0.17	3.86 ± 0.91	2
B: Solid–liquid ratio (*w*/*v*)	2.86 ± 0.13	9.84 ± 0.98	9.64 ± 0.09	7.23 ± 0.87	1
C: Temperature (°C)	8.83 ± 0.97	6.10 ± 0.12	7.42 ± 0.1	2.74 ± 1.2	3
D: Tween (g/L)	7.37 ± 0.1	6.18 ± 0.29	8.82 ± 0.99	2.64 ± 1.26	4

**Table 3:** Infrared spectrum analysis of cotton straw lignin after degradation.

Infrared Spectrum Analysis [27]		Relative Intensities of Absorption Peaks Ai/A1508	
Peak/cm^−1^	Functional group stretching	WT	OE L1-4
3435	O-H stretching in hydroxyl groups	1.66	1.55
2924	C-H stretching in methyl and methyene groups	0.95	0.76
1720	C=O stretching in unconjugated ketone	0.97	0.97
1635	C=O stretching in conjugated aryl ketene of carbonyl groups	1.14	1.12
1595	Aromatic skeletal vibrations plus C=O stretching	1.10	1.11
1506	Aromatic skeletal vibrations	0.99	0.99
1460	C-H deformation in methyl	1.02	1.02
1419	aromatic skeletal combined with C-H in plane stretching	1.02	1.00
1330	Condensation of guaiacyl unit and syringyl unit, syringyl unit and CH_2_ bending stretching	0.96	0.94
1267	G ring plus C=O stretching	1.10	1.15
1218	Aromatic C-O stretching (S units)	1.05	1.09
1128	Aromatic C-H in plane deformations	1.14	1.18
1033	Aromatic C-H in-plane deformation plus C-O deform. In primary alcohols plus C=O stretching	1.03	0.99

**Table 4:** ^1^H-NMR analysis of acetylated lignin from cotton straw medium after degradation.

Chemicalshift Region (δ)	Type of Protons [29]	Percentage of Protons (%)	
		WT	OE L1-4
7.1–6.8	Aromatic protons in guaiacyl units	0.68	0.64
6.8–6.3	Aromatic protons in syringyl units	0.35	0.45
6.2–5.8	Hα of β-O-4 and β-1 structures	0.26	0.31
5.2–4.9	Hydrocarbon protons	0.08	0.17
4.9–4.4	Hγ and H_β_ of β-O-4 structures	0.81	1.11
4.4–4.1	Hα of β-β structures	0.80	0.71
4.1–2.8	H of methoxyl groups	4.83	4.92
2.7–2.2	H of aromatic acetates	1.12	1.52
2.2–1.7	H of aliphatic acetates	4.82	4.16
1.7–1.5	Hydrocarbon protons	0.55	0.37

**Table 5:** Factors and levels of orthogonal test.

Level	Straw Particle Size (mm)	Solid–Liquid Ratio (*w*/*v*)	Temperature (°C)	Tween Content (g/L)
1	0.50	1:2	26	0.15
2	0.75	1:3	27	0.20
3	1.00	1:4	28	0.25

**Table 6:** Primer sequence used in this study.

Primer Name	Primer Sequence (5′-3′)
Lac1-F	ATGCGCACATTCTCCCGCTTTCTC
Lac1-R	CTAGGTTGGGAGCAAACCGCCTTTTTCC
Lac1-qF	CGGTACATCTTAGCACCCAATG
Lac1-qR	GGACAGGGCTCGCTGGTT
cyph-F	GACATTGCTATCGACTCCCAG
cyph-R	GAAATTCCTTGCAGTCTTGGG
Pogpd-4F	CGGAATTCTCTGGAATCGTTATCTCGGT
Pogpd-1R	CGGGATCCCGTGGACAGGCTTTTGGGAATA
Lac1-eukF	GAAGATCTGATGCGCACATTCTCCCGCTTTCTC
Lac1-eukR	GGACTAGTGGTTGGGAGCAAACCGCCTTTTTC
GUS-F	GTCCTGTAGAAACCCCAACCCGTGA
GUS-R	TTTGCCTCCCTGCTGCGGTTTTTCA

## Data Availability

Not applicable.
